# Ilizarov’s technique: A pioneer’s vision

**DOI:** 10.3126/nje.v15i1.72248

**Published:** 2025-09-02

**Authors:** Shantimoy Banerjee, Indrajit Banerjee, Jared Robinson, Indraneel Banerjee

**Affiliations:** 1 Jawaharlal Nehru Memorial (JNM) Hospital, Kalyani, India; 2,Sir Seewoosagur Ramgoolam Medical College, Belle Rive, Mauritius; 3 Joe Morolong Memorial Hospital, Department of Medicine, Vryburg, South Africa; 4 Penn Highlands Healthcare, Pennsylvania, United States of America

## To The Editor:

Orthopedic surgical cases are among the most common entering hospitals. Orthopedic surgery is a surgical discipline which is diverse in nature and requires a high level of skill and training as well as specialized equipment and surgical teams [1]. The majority of orthopedic surgeries are costly and are often performed by super-specialized surgeons in that particular anatomical region or field. This makes performing orthopedic surgeries in resource limited regions a challenge. One such procedure which has been pioneered by Dr. S.M Banerjee in rural areas such as Kalyani, West Bengal, India is the use of the Ilizarov technique [2].

## History

This technique was developed in the Soviet Union by a physician named Gavriil Abramovich Ilizarov, who successfully treated his first patient with this method in 1954 [3]. The patient suffered from a tibial non-union. Ilizarov inadvertently discovered the principle of distraction osteogenesis, on observing a callus formation in a patient who had accidentally distracted the frame as opposed to compressing it [4, 5]. This new technique allowed multiple nonunion cases to be surgically corrected and thus quickly fueled the popularity of both Dr. lizarov and his technique. Although Ilizarov had no formal orthopedic training his technique has revolutionized corrective surgery and it is both still applicable, relevant and in use to this day [6]. The Ilizarov technique is a minimally invasive and limb saving technique which is employed via the use of multiple trans-osseous pins and an external ring fixator (Ilizarov fixator). It is based on the physiological theory and phenomenon of distraction osteogenesis and it is almost limitless in its applications [7]. It can be used to treat major trauma such as comminuted fractures, to limb lengthening procedures, limb defects, limb deformities, non-unions and even the correction of clubfoot [8, 9].

## Surgical Procedure

The procedure has multiple variations and adaptations as it has been in use since the 1960’s. The basic principles of the procedure for the most part however remain the same. The Procedure is based on distraction osteogenesis, continual external tension lengthens the bone and or corrects deformities. The external ring fixator holds multiple intraosseous pins which can provide compression, distraction, angulation and or rotation, depending on the case. The pins are driven from the side which is nearer to sensitive structures, thus ensuring they do not damage any neurovascular bundles. Bones between the segments of elongation may be separated by corticotomy and distraction through the pins is usually done at 1mm/day in four intervals at 0.25mm per session. After the desired distraction has taken place and the desired length is achieved the consolidation phase is initiated. The entire procedure can be subdivided into 5 phases. Phase 1: Application of the fixator, usually for 1 week. Phase 2: Distraction or compression for 1 to 4 months. Phase 3: Immobilization and fixation, most commonly double the period of phase 2. Phase 4: Frame dynamization (cease compression/distraction). Phase 5: Immobilization with a brace or cast [10-12].

## Pioneers of the technique

Dr. S. M. Banerjee became a pioneer in the Ilizarov technique, in resource-constrained rural settings over 40 years ago. Performing the Ilizarov technique in such remote settings is both a challenging and indeed praiseworthy accomplishment. Dr. Banerjee attended an International workshop on the Ilizarov technique in Kolkata in the late 1980s and was fascinated and inspired by the results and its endless applications. This became the catalyst for him to perform such surgery in rural West Bengal.

Dr. S. M. Banerjee performed his first case on a young 18-year-old male patient with an infected tibia with massive bone loss due to a road traffic accident who was admitted at Jawaharlal Nehru Hospital, Kalyani waiting for a below knee amputation. Dr. S. M. Banerjee wanted to salvage the limb so he applied the Ilizarov technique. He was assisted by a young doctor from Kolkata as first assist. The procedure was successful. Unfortunately, the patient suffered a severe secondary hemorrhage post-operatively and after 3 weeks, the limb could not be saved. This did not however stop him from applying this method in his future days. Dr. S. M. Banerjee started applying this technique in various cases ranging from fresh fractures of the tibia to several infected tibias with bone loss of approximately 3 inches. Dr. S. M. Banerjee went so far as to extend the use of the surgery into tibial fractures in children which showcased excellent results. Dr. S. M. Banerjee performed the Ilizarov technique in approximately twenty five patients with an overall success rate of 80%. Dr. S. M. Banerjee couldn’t continue this procedure as it is expensive, time-consuming and needs a dedicated team and requires more patience, all of which are not feasible in resource limited settings.

## Expert opinion

The Ilizarov technique is a highly diverse and multipurpose surgery which allows for the rectification of complex and challenging cases. The ability for a single surgeon to be able to correct a plethora of conditions with the slight modification of the technique is highly valued in resource limited rural settings where the likelihood and means of such patients to reach a tertiary center is near impossible. Although the procedure may be a fraction more costly and does require a dedicated staff, the near endless applicability of the technique offsets these challenges. All rural based orthopaedic surgeons should both be trained and well versed in the Ilizarov method so as to ensure that even the rural populous have access to a better level of care without having to undergo the trauma of being removed from their locality in order to undergo a procedure at a higher institute. With further aid and support from both local and international authorities, the disparities between the rich and poor as well as health inequality can be minimized via the employment of such a technique in the rural setting.

S.M. Banerjee, Department of Orthopaedics, Jawaharlal Nehru Memorial (JNM) Hospital, Kalyani, India

Indrajit Banerjee, Sir Seewoosagur Ramgoolam Medical College, Belle Rive, Mauritius

Jared Robinson, Joe Morolong Memorial Hospital, Department of Medicine, Vryburg, South Africa

Indraneel Banerjee, Urologist and Robotic Surgeon, Penn Highlands Healthcare, Pennsylvania, United States of America

Dated: 10 March, 2025
